# Non-quantitative adjustment of offspring sex ratios in pollinating fig wasps

**DOI:** 10.1038/srep13057

**Published:** 2015-08-21

**Authors:** Rui-Wu Wang, Bao-Fa Sun, Jun-Zhou He, Derek W. Dunn

**Affiliations:** 1 Center for Ecological and Environmental Sciences, Northwestern Polytechnical University, Xi’an, 710072, China; 2 Disease Genomics and Individualized Medicine Laboratory, Beijing Institute of Genomics, Chinese Academy of Sciences, Beijing 100101, China; 3 Statistics and Mathematics College, Yunnan University of Finance and Economics, Kunming, Yunnan, 650221, China; 4 State Key Laboratory of Genetic Resources and Evolution, Kunming Institute of Zoology, Chinese Academy of Science, Kunming, Yunnan, 650223.

## Abstract

Fig wasp is one of the most well known model systems in examining whether or not the parents could adjust their offspring sex ratio to maximize their gene frequency transmission in next generations. Our manipulative experiments showed that, in all of the five pollinator wasps of figs (Agaonidae) that have different averages of foundress numbers per syconium, almost the same proportions of male offspring are produced in the experiment that foundresses deposit one hour then are killed with ether (66.1%–70.1%) and over the lifespan of each foundress (14.0%–21.0%). The foundresses tend to deposit their male eggs prior to female eggs. The observed increase in the proportion of male offspring as a function of foundress number results from density-dependent interference competition among the foundresses. These results showed that the selection of gene frequency transmission through the behavioral adjustment in the evolution of sex ratio does not exist in these five fig wasps. The results here implied that genetic adjustment mechanisms of the sex ratio of fig wasps can only be triggered to be on or off and that the foundresses can not quantitatively adjust their sex ratio according to increased environmental selection pressure.

The evolution of sex ratio is regarded as a fundamental characteristic of particular organisms. The fundamental theoretical development of Fisher showed that, in a large randomly-mating population, frequency-dependent selection would lead to equal parental investment in the two sexes resulting in the evolution of a 1:1 sex ratio because of the trade-off between male and female investments^1^. Hamilton’ revelatory theory proposed that organisms could adjust their offspring to a female-biased sex ratio in isolated sub-populations (i.e. in a limited-dispersal colony) because of inbreeding and Local Mate Competition (LMC) in which the male offspring would contribute less to the population^2^. Many studies have suggested that a female-biased sex ratio can be adjusted by parents responding to either inbreeding, LMC intensity, or other environmental factors, which can maximize the fitness of the parents^3^,^4^.

A remained question on above theories is what mechanisms could help the parents in “recognizing or calculating” how many male or female offspring produced by their neighbors, or by themselves, if parents were able to quantitatively adjust the sex ratio of their offspring to maximize their fitness according to the intensity of inbreeding and LMC. In addition, theories of quantitative adjustment of sex ratio have not considered that the recognition or calculation cost might increase with an increase in neighbor size or offspring population. In unpredictable environments, such costs could exceed the benefits gained by foundresses due to the adjustment of the sex ratio of their offspring^3^, especially when frequent migration among the reproducing females occurs^5^. In unpredictable environments, most individuals might change their reproduction strategy or produce different numbers of offspring under different situations and reproductive females might not be able to predict how many offspring their neighbors or themselves might produce. It may therefore be impossible for such females to predict the “best sex ratio” for their offspring in indeterminate environmental conditions^6^.

In this research, using one of the most well documented sex ratio model systems of fig wasps, we re-examined whether or not parents (i.e., foundresses in fig wasp science community) could adjust their offspring ratios under different conditions of inbreeding and LMC intensity. Since Hamilton in 1979 pointed out that fig wasps might provide evidence for his theory that inbreeding and LMC might lead to a female-biased sex ratio^7^, empirical data have demonstrated that fig wasps are able to adjust the sex ratio of their offspring, responding to a variation in foundress numbers within a brood (i.e. responding to LMC) or to averaged foundress numbers (i.e. responding to inbreeding value)^8^,^9^,^10^,^11^,^12^. However, the recent empirical data, especially in the manipulative experiments, showed that quantitative adjustment triggered by inbreeding and LMC can not sufficiently explain the observed sex ratio of fig wasps^3^,^10^,^13^,^14^,^15^.

Theoretically, the proportion of male increase in wasp offspring with an increase in foundress numbers might also result from the density-dependent interference among foundresses^13^,^15^,^16^, but not because of positive adjustments of foundresses to the sex ratios of offspring. Many Hymenoptera females lay male eggs prior to female eggs. An increase in the proportion of male offspring as a function of foundress number may therefore be a result of a decrease in the time allocated to female egg deposition. The decreased number of oviposition sites or increased interference competition among the foundresses, which mainly negatively affect the female egg deposition, might therefore increase the male proportion in offspring number^14^,^15^.

## Materials

### (a) Sampling sites

Our work was carried out in Xishuangbanna, Yunnan, China. Our field site is in the south of the Yunnan province (21°41′N, 101°25′E), where the altitude is approximately 600 m and the climate includes a wet and dry season. In Xishuangbanna, the wet season lasts from May to October. Our field studies were carried out in or around the Xishuangbanna Tropical Botanic Garden. The field observations or experiments have gotten a permission of Xishuangbanna Tropical Botanic Garden, if the related observations or experiments are carried out on their grounds.

### (b) Study species

For the manipulative experiments in Xishangbanna, China, we used the following pollinator wasps and hosts: *Ceratosolen fusciceps* hosted by *Ficus racemosa*; *C. solmsi* hosted by *F. hispida*; *Eupristina koningsbergeri* hosted by *F. benjamina*; *C. gravelyi* hosted by *F. semicordata* and *C. emarginatus* hosted by *Ficus auriculata*. All of the host plant species of these pollinator wasps are commonly distributed in Xishangbanna and are easy to obtain in the Xishuangbanna Tropical Botanic Garden or at sites around the garden.

## Methods

### (a) Oviposition sequence

When the figs were receptive to oviposition, we located other figs just prior to wasp release and enclosed them in nylon bags (i.e. ‘bagged them’). On the day following wasp emergence we allowed one foundress from the nylon bags to enter each of the receptive fruits. The figs were then ‘bagged’ again to prevent further pollinator entries or parasitism by non-pollinating fig wasps and left to develop. To determine the sex ratio of the offspring that were deposited during specific periods of an oviposition sequence, we killed foundresses at different time intervals after they had entered the figs, by injecting ether into the treated syconia. In each syconium, we injected 0.05 ml for *F. racemosa*, 0.02 ml for *F. benjamina*, 0.04 ml for *F. hispida*, 0.04 ml for *F. semicordata*, or 0.3 ml for *Ficus auriculata* with 1, 2, 4, 6, 8 hour intervals respectively. Preliminary tests indicated that such dosages disabled the foundresses for egg deposition within three minutes (When the figs were cut open, all the foundresses were dead). One set of treatment was left as a control, allowing the fig wasps to oviposit naturally.

Because the syconia would be frequently aborted if they were not sufficiently pollinated or ovipoisited^17^,^18^,^19^, we introduced two extra foundresses from which the ovipositors were cut transversely with a scalpel. Our previous studies validated that these foundresses could pollinate and their life activity is little affected, but would not be able to oviposit any eggs into the female fig flowers (see Wang *et al.* in 2010 for details of this treatment)^17^,^18^,^19^. These extra two foundresses were introduced after the experimental foundress died (one day later) and such experimental treatment will avoid the potential competition among these foundresses. Through this method, most of the treated syconia could develop to the mature stage and adult wasps could be collected from the treated syconia. By counting adult wasps from differently-treated syconia, we could identify whether the fig wasps tended to lay their male eggs before the female eggs.

### (b) Interference identification

To examine whether the interference competition among the fig wasps could discriminatively affect male egg and female egg deposition, we simultaneously introduced foundresses into the receptive syconium cavities in a set experiments but sequentially in another set of experiments. In simultaneous introduction, the foundresses are introduced into the receptive syconium cavities within 30 minutes; in contrast, foundresses sequentially are introduced the into receptive syconium cavities with time intervals: four tests were conducted in which two, five, seven and nine foundresses were separately introduced for each treatment. In the two-foundress treatment we introduced the first and second foundresses at 9 am on two consecutive days, respectively. In the other three treatments with respectively five, seven and nine foundresses in total, considering their lifespan and receptive duration time, we introduced two on the first day at 9 am and then, sequentially, two foundresses, or one foundress if only one was left, every three to four hours in the day time or 10 to12 hours in the evening. Such comparison experiments on interference competition were conducted using the following four pollinator species: *C. fusciceps* hosted by *F. racemosa, C. solmsi* hosted by *F. hispida, Eupristina koningsbergeri* hosted by *F. benjamina, C. gravelyi* hosted by *F. semicordata* and *C. emarqinatus* hosted by *F. auriculata*.

**Figure 1:**
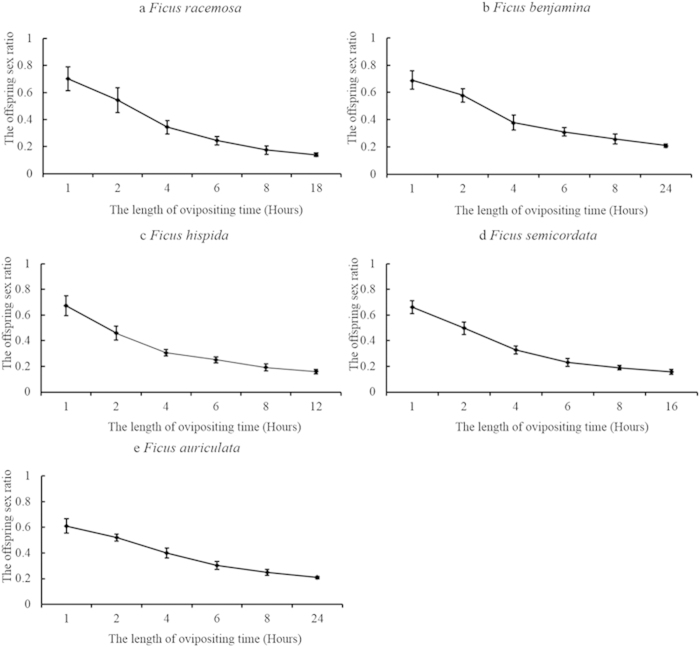
Offspring sex ratio (male offspring/total offspring number) of pollinator wasps at different ovipositing periods in five fig species (mean ± SD). (**a**) *F. racemosa* pollinated by *C. fusciceps*; (**b**) *F. hispida* pollinated by *C. solmsi*; (**c**) *F. benjamina* pollinated by *E. koningsbergeri*; (**d**) *F. semicordata* pollinated by *C. gravelyi*; (**e**) *F. auriculata* pollinated by *C. emarginatus*. The sample size N ≥ 20 in each type of the experiments.

The interference competition impact has been showed to be density-dependent and the interference competition that decreases the female egg deposition will be non-linearly amplified as a function of foundress number^15^,^16^. The sex ratio would be expected to increase to over the 50% male offspring level after the foundress number reaches a threshold in which interference competition among the foundresses was very high and the sex ratio should be same ratio in the first minute/hour of egg deposition in each foundress (i.e. male offspring proportion will be about 70% when the foundress number is very high, see Fig. 1). We therefore designed the simultaneous introduction of pollinators of *F. racemosa* with 1, 3, 5, 7, 9, 15, 20, 30, 40, 70 foundresses per syconium to examine whether the sex ratio would increase to over 50% after the foundress number had reached a certain threshold.

In all of the above manipulative experiments, we selected two trees for each species from two sample sites to conduct these experiments. These two sample sites were usually more than one kilometer apart. In each tree, we sampled 4–6 syconia for each type of experiment. In each type of these experiments, the sample size N is 20 or a little larger than 20.

## Results

**Figure 2:**
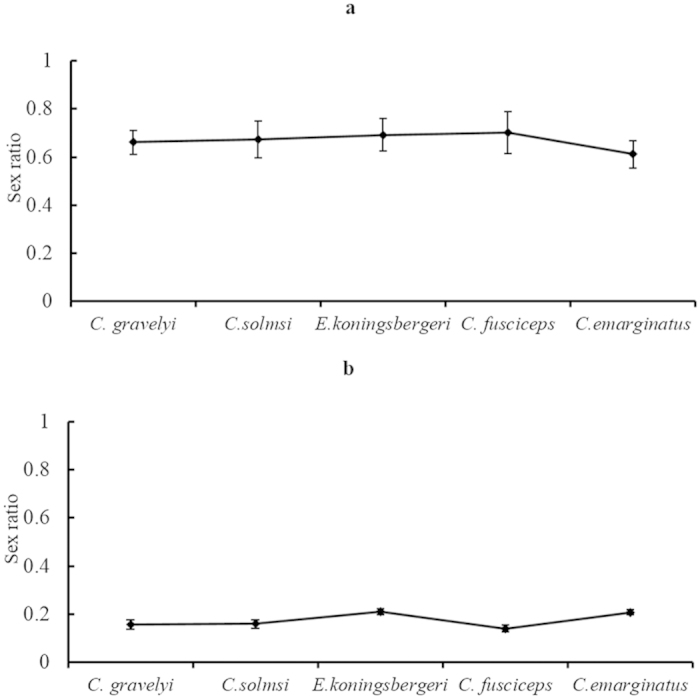
The mean offspring sex ratio (male offspring/total offspring number) of single foundress in five fig wasp species (mean ± SD): (**a**) deposition within the first one hour; (**b**) deposition over their lifespan. The average foundress number per syconium differs greatly among the five species in literatures^46^,^47^,^48^: *Ceratosolen gravelyi*:1.60 ± 0.10, n = 168; *Ceratosolen solmsi*: 2.08 ± 1.65, N = 182; *Eupristina koningsbergeri*: 2.73 ± 1.56, N = 90; *C. fusciceps*: 4.50 ± 4.16, N = 1590; *Ceratosolen emarginatus*: 8.62 ± 16.21, N = 55. The sample size N ≥ 20 in each type of the experiments.

Results indicated that, as was the case for a number of other reports on fig wasp reproduction^10^,^20^,^21^,^22^,^23^, all of the pollinator wasps (i.e. *Ceratosolen fusciceps* hosted by *Ficus racemosa, Ceratosolen solmsi* hosted by *F. hispida, Eupristina koningsbergeri* hosted by *F. benjamina, Ceratosolen gravelyi* hosted by *F. semicordata, C. emarginatus* hosted by *F. auriculata*) tend to lay their male eggs prior to the female eggs (Fig. 1). In all of these treated pollinator species, the male offspring proportion per foundress was 66.1%–70.1%, within the first hour of deposition. The proportion of male offspring for each foundress decrease with an increase in the length of egg deposition time and the proportion of male offspring of each foundress over its lifespan range from 14.0% to 21.0% in the controlled experiments. The male offspring proportions within the first one hour of egg deposition were, however, very similar in these five treated pollinator species (Fig. 2). In these five pollinator species, the averaged foundress numbers per syconium vary from 1.60 ± 0.10 (*E. koningsbergeri*)to 8.62 ± 16.21 (*C. emarginatus*) (mean ± SD), however, sex ratio of one foundress in the controlled experiment showed that the male offspring proportion do not increase in the species with higher averaged foundress number (Fig. 2b). The data in these five species do not conform to that higher inbreeding value (i.e., lower averaged foundress number per syconium) will lead to lower male proportion in wasp offspring predicted in the Hamilton’s sex ratio evolution theory (see the appendix).

**Figure 3:**
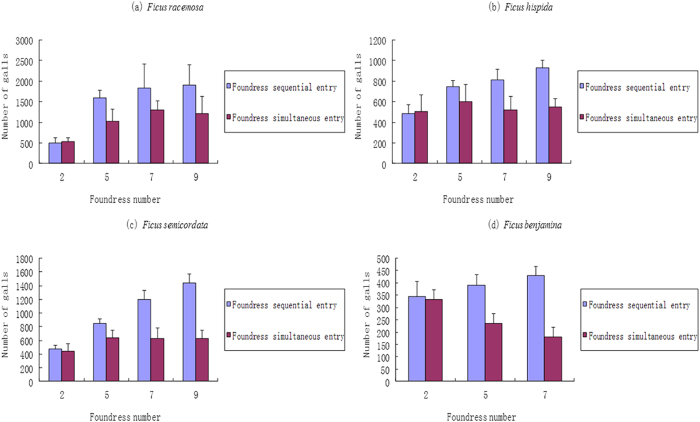
The galled flower numbers (egg number) in the experiments of simultaneous introduction of foundresses and the experiments of sequential introduction of fundresses with different foundress number in four species (mean ± SD). The sample size N ≥ 20 in each type of the experiments.

The experiment results showed, when nine foundresses were compared, that the sequential introduction experiments have higher averaged offspring numbers than in simultaneous introduction (Fig. 3). The mean comparison results are: in *F. benjamina* (foundress number = 7), t = 21.231, df = 38, *P* < 0.001; in *F. semicordata*, t = 15.420, df = 38, *P* < 0.001; in *F. hispida*, t = 20.626, df = 38, *P* < 0.001; in *Ficus racemosa*, t = 4.56, df = 39, *P* < 0.001. Interference competition is more significant when the foundress number is relatively high and the interference competition is weak or does not exist when the foundress number is low. For the sake of brevity, we do not show all the comparison results in this paper.

**Figure 4:**
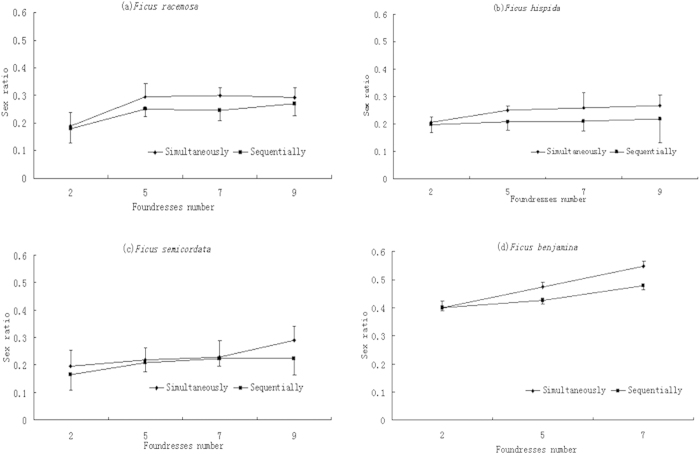
Sex ratio under different introduction treatments (mean ± SD). In four fig species, if the pollinator wasps are simultaneously introduced into receptive syconia, the male offspring ratio will be higher than for sequential introduction when interference competition among the foundresses is reduced. The sample size N ≥ 20 in each type of the experiments.

**Figure 5:**
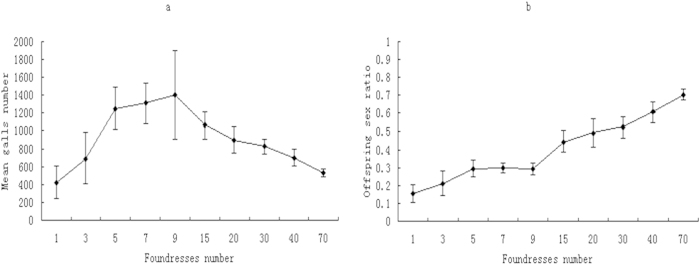
Offspring number or sex ratio as a function of foundress number. (**a**) Total average numbers of egg deposited (measured as the number of galled flowers) vs foundress numbers and (**b**) the sex ratio vs foundress numbers in *F. racemosa*. Results indicate that total egg deposition will decrease after foundress numbers reach a threshold and the male offspring sex ratio will increase as a function of foundress number. The male offspring sex ratio does not asymptotically reach 50%, but increases to above the 50% level to 70% of which is the male offspring sex ratio in the first hour egg deposition. The sample size N ≥ 20 in each type of the experiments.

Interference competition among the foundresses results in decreases of female egg deposition. Sequence introduction can decrease the interference competition among the foundressses and the male offspring ratio in sequence introduction is lower than in simultaneous introduction, when the number of foundresses is high (Fig. 4). Including the syconium size as covariates, the sex ratio comparison results between sequence introduction and simultaneous introduction with 9 foundresses are: in *F. racemosa, F*_1, 40_ = 1.14, *P* > 0.05; in *F. hispida, F*_1, 39_ = 4.21, *P *< 0.05; in *F. semicordata, F*_1, 39_ = 4.68, *P* < 0.01; in *F. benjamina* (7 foundresses), *F*_1, 39_ = 46.98, *P *< 0.001. In *F. racemosa*, when the foundresses are manually increased due to simultaneous introduction, the male offspring ratio increases significantly as a function of the number of foundresses and can reach 70.1%. The male offspring proportion of the pollinators of *F. racemsoa* does not increase to an asymptote of 50% predicted in the Hamilton’s sex ratio evolution theory (see appendix). The averaged male offspring proportion with higher foundress number is significantly higher than in the treatments with lower foundress number (*F*_9, 200_ = 173.17, P < 0.001), (Fig. 5).

In *F. racemsoa,* interference competition intensity among the foundresses is greatly affected by the syconium size or the availability of female flowers per syconium. In the comparison experiments in which nine foundresses were simultaneously introduced into receptive syconia, the total number of wasp offspring in the syconia with larger diameters (mean diameter 50.26 ± 4.36 mm, N = 20) was 1403.15 ± 491.43 averaged total galls, but in the smaller syconia (of mean diameter 41.15 ± 4.73 mm, N = 20), the mean number of galls was 1026.95 ± 215.78. In *F. racemsoa*, the mean egg deposition efficiency (gall number per foundress) positively correlated with the syconium diameter in the experiment with nine foundresses that were simultaneously introduced (N = 40, r = 0.47, *P* < 0.05). The oviposition efficiency of foundresses was also significantly correlated with the total number of female flowers (N = 40, r = 0.53, *P* < 0.001). The larger the syconium size, the higher the number of total female flowers. This will lead to less interference competition among foundresses. Thus lower negative impacts on female egg deposition can be expected in syconia with larger diameter sizes, or with higher total numbers of female flowers (see Supplementary Information).

## Discussion

It has long been believed that the parents can adjust the sex ratio of their offspring in response to environmental conditions in order to maximize their biological fitness since work by Hamilton in 1967 and Trivers-Willard model in 1973^24^. However, the meta-analysis by West and Sheldon in 2002^3^ indicated that evidence of such environment-oriented sex adjustment is not always convincing. Empirical data and/or observations of fig wasps also showed different, or even contradictory, results on whether the offspring sex ratio could be adjusted responding to inbreeding and LMC variation. Some empirical observations of fig wasp species well conform to the theoretical predictions outlined in some studies (e.g.^8^,^9^,^12^,^25^), but others showed that the sex ratio (male proportion) may greatly deviate from theoretical predictions. Evidence in much of the literatures indicate that the sex ratios of fig wasps are greatly affected by non-inbreeding, or non-LMC factors (e.g., clutch size, male egg deposition sequence, interference competition among foundresses and other factors)^10^,^13^,^14^,^18^,^20^,^23^,^26^,^27^.

Our examination, using manipulative experiments on fig wasps in Xishuangbanna of China, disclosed that observed increases in the proportion of male offspring as a function of foundress number was not a result of adjustments to sex ratio by parents responding to inbreeding and LMC selective pressure, but from interference competition among foundresses. Because the wasps tend to deposit their male egg (unfertilized eggs) prior to female eggs (Fig. 1), interference competition, which will decrease the total egg deposition of each foundress, will result in a decrease of female egg deposition and will increase the proportion of males in the offspring. Interference competition intensity is strongly influenced by foundress number, the cavity size of the syconium, or the availability of total female flowers. These factors explain why the sex ratio of pollinator wasps varies according to clutch size or syconium size as reported in some literatures^10^,^14^,^18^,^20^.

Average foundress numbers vary in different species^28^,^29^. It can be expected that evolutionary inbreeding pressure will differ among pollinator species (see Fig. 2 and 8,23,25,30,31,32. In such species, however, the proportion of males in the wasp offspring over an entire lifespan of each foundress does not increase with increases in the average number of foundresses per syconium. The deviation of male wasp offspring among pollinator species may result from lifespan differences in the treated wasps, because the lifespans of pollinator wasps differ under different environmental conditions. For example, temperature and humidity greatly affect the lifespan of wasps e.g.^15^,^33^,^34^). The proportion of male eggs deposited in the first hour of deposition is almost the same and lifespan variation will greatly affect the female egg deposition. Therefore, the sex ratio will be sensitive to the lifespan of foundresses. Data obtained in our experiments indicated that fig wasps are not be able to adjust their sex ratio quantitatively in both the evolutionary scale (i.e. adjustment according to the inbreeding coefficient) and in the temporal/behavioral scale (i.e. adjustment according to LMC).

Our results imply that genetic regulation of these five fig wasp parents that determines the sex ratios of offspring might be due to an on-off switch mechanism^35^,^36^,^37^. Increases in environmental selective pressure can not quantitatively be triggered by genetic regulation mechanisms, but can be turned on or off once environmental selective pressure reaches certain thresholds. The sex ratio adjustment can be triggered to be either ‘on’ or ‘off’. Although such sex ratio adjustment mechanisms have been implied in paper of Whiting in 1943^35^ and such on-off switch mechanism of gene expression have been widely demonstrated in genetics [e.g.^28^,^38^,^39^], the study of such mechanisms has been neglected in fig wasp science community^24^. Our empirical data implies that such sex ratio adjustment mechanisms might be true in these five fig wasps in Xishuangbanna, China.

The remaining problems of sex ratio allocation in fig wasps relate to the dynamics that drive some species to deposit male eggs prior to female eggs and have higher average numbers of female offspring than males over the lifespan of each individual for some species, whereas some species deposit male and female eggs in a random fashion, resulting in sex ratios close to 1:1. In some fig wasp communities pollinator wasps tend to deposit male eggs prior to female eggs, which results in a female-biased sex ratio, whereas some non-pollinators randomly deposit their egg without sex sequence discrimination^18^. In the fig wasp community supported by their host species, both inbreeding and LMC value of pollinator wasps should be higher than would be the case for non-pollinators because the pollinators usually enter syconium cavities to deposit their eggs and the offspring pollinator fig wasps usually mate within the limited population before they exit the fruit cavities^18^,^34^,^40^,^41^. In empirical observations, the pollinator fig wasps are much more female biased than non-pollinator wasps [18, 27]. The genetic adjustment mechanism in pollinator fig wasps therefore might be triggered by the selection pressure of inbreeding and LMC through the mechanism of male-first egg deposition.

Differences in mating systems among the wasps during egg deposition might be another possible dynamic that triggers the genetic regulation of sex in fig wasps. Most adult pollinator fig wasps mate within a limited space (syconium cavities) before they exit their natal syconia^18^,^38^. These mating systems might lead to quantitative or qualitative hormone variations that could directly affect the genetic regulation mechanism and whether such mechanisms are triggered as ‘on’ or ‘off’^39^,^42^. Research reported in the scientific literatures have indicated that, if the wasps mated outside of the syconium, the offspring exhibited lower levels of female bias^18^,^38^,^43^. Differences in mating systems might be a alternative potential mechanism explaining why some fig wasps are less female-biased than others^38^,^44^,^45^ or even why some fig wasps present sex ratios close to 1:1^18^.

## Additional Information

**How to cite this article**: Wang, R.-W. *et al.* Non-quantitative adjustment of offspring sex ratios in pollinating fig wasps. *Sci. Rep.* **5**, 13057; doi: 10.1038/srep13057 (2015).

## Supplementary Material

Supplementary Information
