# Supplementary material for: Non-quantitative adjustment of offspring sex ratios in pollinating fig wasps
Source: Sci Rep. 2015 Aug 21;5:13057. doi: 10.1038/srep13057 (PMC4543974; doi:10.1038/srep13057)
Supplement: Supplementary Information [file srep13057-s1.docx]

**Additional information**

Non-quantitative adjustment of offspring sex ratios in pollinating fig wasp

Rui-Wu Wang ^1, 4^, Bao-Fa Sun ^2^, Jun-Zhou He ^3^, Derek W. Dunn ^4, 1^

Running title: sex ratio of fig wasps

1. Center for Ecological and Environmental Sciences, Northwestern Polytechnical University, Xi’an, 710072, China.

2. Disease Genomics and Individualized Medicine Laboratory, Beijing Institute of Genomics, Chinese Academy of Sciences, Beijing 100101, China.

3. Statistics and Mathematics College, Yunnan University of Finance and Economics, Kunming, Yunnan, 650221, China.

4. State Key Laboratory of Genetic Resources and Evolution, Kunming Institute of Zoology, Chinese Academy of Science, Kunming, Yunnan, 650223.

Correspondence and requests for materials to Rui Wu Wang.

Authors for Correspondence

[ruiwukiz@hotmail.com](mailto:ruiwukiz@hotmail.com)

Appendix: The theoretical pattern of sex ratio of Hamilton’s sex ratio evolution theory (see Herre 1985).

The proportion of sib-mating in a population determines the equilibrium level of inbreeding, which here will be taken as the probability that an allele at a given locus that a daughter receives from her father is identical by direct descent (idd) to alleles of her mother. In order to calculate that proportion, consider the population of mated mothers to be composed of four types: $a_{1}$ is the proportion of mothers somatically homozygous, mated with idd sperm; $a_{2}$ is the proportion of mothers somatically homozygous, unrelated sperm; $a_{3}$ is the proportion of mothers somatically heterozygous, mated with idd sperm possessing alleles (idd) to one of the two maternal alleles; and $a_{4}$ is the proportion of mothers somatically heterozygous, mated with nonrelated sperm. Given the following assumption ---(i) random mating within broods, (ii) no inbreeding depression, (iii) no relation among cofounderses of a given brood, (iv) equal contribution of offspring at equal sex ratios among cofounderses of a given brood, and (v) equal total of females dispersing from all broods (16)-----then the equilibrium proportion of each maternal type is given in terms of the harmonic mean number of foundresses per brood(15), $n$, by the following system of recursion equations: $a_{1}=(1/n)(a_{1})+(1/4n)(a_{3})$;

$a_{2}=[(n-1)/n](a_{1})+[(2n-1)/4n](a_{3})$;$a_{3}=(1/n)(a_{2})+(1/2n)(a_{3})+(1/2n)(a_{4})$;

$a_{4}=[(n-1)/n](a_{2})+[(n-1)/2n](a_{3})+[(2n-1)/2n](a_{4})$ and $a_{1}+a_{2}+a_{3}+a_{4}=1$

At equilibrium, the proportion of daughters with both somatic alleles shared (idd) with their mothers is $a_{1}+a_{3}=1/(2n-1)$. The standard inbreeding coefficient is $F=a_{1}+a_{2}=1/(4n-3)$. Therefore, at equilibrium, the average daughter shares $1+1/(2n-1)=2n/(2n-1)$ alleles (idd) with her mothers while a son shares one. Computer simulations show the calculation of level of inbreeding to be robust in the face varying foundress numbers through time and wide departures from the assumptions of equal contribution at equal sex ratio to brood. Multiple mating also do not affect the equilibrium level of inbreeding. The inbreeding dependent relatedness of daughters, $2n/(2n-1)$, and that of sons, one, to mothers is then used to weight the relatedness of broods of grandprogeny produced by sib crosses, daughter outcrosses, and son outcrosses. The expected frequency of each of these types of mating events is calculated with the proportion and sex ratio of the offspring a given mother contributes to a brood and the proportion and sex ratio of the offspring that other mothers contribute to that brood. The weighted frequencies are summed and differentiated with respect to the sex ratio of given mother’s offspring, $p$. the maximum is determined, and solving for $p$ produces a general formula for predicting brood sex ratios that maximize a mother’s efficiency in exploiting a brood as a resource for propagating her alleles, given any level of inbreeding in the population, any proportion of the brood that she contributes, and any sex ratio of the offspring of other mothers

$$p=\frac{[\frac{(4n-1)(1-m)q}{2n-1}]^{1/2}-\frac{(4n-1)(1-m)q}{2n-1}}{\frac{(4n-1)m}{2n-1}}$$

Where $p$ is the proportion of male in a given foundress’ offspring, $m$is the proportion of a given brood a given mother contributes, $q$ is the proportion of males in the rest of the brood, and $n$ is the harmonic mean foundress number in the population. The Hamilton and Taylor-Bulmer equations for predicting haplodiploid sex ratios are special cases of this equation, derivable by setting $m=1/n$and $q=p$ (that is, by assuming fixed inbreeding for fixed intensities of LMC). The Werren equation that can predict sex ratios given variable relative contributions to broods by two foundresses and that did not take into account effect of inbreeding can be derived by setting $(4n-1)/(2n-1)=2$ and dividing through by $(1-m)$. Setting $(4n-1)/(2n-1)=2$ makes Eq. 1 appropriate for diploid organisms. By setting equal the contributions of all cofoundresses to any given brood, another equation for *p* emerges

$$(1-m)(2n-1)/(4n-1)$$

This equation is used to generate the predicted sex ratios resulting from the interplay of the sex ratios resulting from the interplay of the effects of inbreeding and LMC in fig wasps (Figs. 6). Notice that the best brood sex ratio, p, for inbred haplodiploid foundresses $[(2n-1)/(4n-1)]<1/2$ is female-biased even in populations to which their contribution of offspring vanishingly small (as m approaches 0, that is, in large outbreeding population) (Fig. 6). Although the condition of inbred mothers in outbred population is unstable, this result points to the fact that inbreeding selects for female-biased sex ratios independently of any effects of LMC or differential group productivity, which has been verified by appropriate simulation.

Reference

Herre, E. A. Sex-Ratio Adjustment in Fig Wasps. *Science* **228**, 896-8 (1985).
